# Efficacy and Safety of Pioglitazone Add‐On in Patients With Type 2 Diabetes Mellitus Inadequately Controlled With Metformin and Dapagliflozin: A Systematic Review and Meta‐Analysis of Randomised Controlled Trials

**DOI:** 10.1002/edm2.70061

**Published:** 2025-05-27

**Authors:** Ubaid Khan, Zuhair Majeed, Muhammad Haris Khan, Ahmed Bostamy Elsnhory, Ahmed Mazen Amin, Anum Nawaz, Ahmed Raza, Hafiz Muhammad Waqas Siddque, Mustafa Turkmani, Mohamed Abuelazm

**Affiliations:** ^1^ Division of Cardiology University of Maryland School of Medicine Baltimore Maryland USA; ^2^ Department of Medicine King Edward Medical University Lahore Pakistan; ^3^ Department of Medicine Saidu Medical College Swat Pakistan; ^4^ Emergency Medicine Department Al‐Bank Al‐Ahly Hospital Cairo Egypt; ^5^ Faculty of Medicine Mansoura University Mansoura Egypt; ^6^ Department of Radiology Pakistan Atomic Energy Commission Hospital Islamabad Pakistan; ^7^ Department of Medicine Services Institute of Medical Sciences Lahore Pakistan; ^8^ Department of Medicine MedStar Health Georgetown University (Baltimore) Program Baltimore Maryland USA; ^9^ Faculty of Medicine Michigan State University East Lansing Michigan USA; ^10^ Department of Internal Medicine McLaren Health Care Oakland Michigan USA; ^11^ Faculty of Medicine Tanta University Tanta Egypt

**Keywords:** dapagliflozin, metformin, pioglitazone, review, type 2 diabetes mellitus

## Abstract

**Background:**

Type 2 diabetes mellitus (T2DM) accounts for over 90% of diabetes cases worldwide. Pioglitazone, a thiazolidinedione, enhances insulin sensitivity by activating PPAR‐γ. Evidence on its efficacy and safety as an add‐on to metformin and SGLT2 inhibitors in inadequately controlled T2DM is limited. This systematic review and meta‐analysis evaluates pioglitazone's role as a third‐line therapy for improving glycaemic control in addition to metformin and Dapagliflozin.

**Methodology:**

We conducted comprehensive searches across PubMed, CENTRAL, WOS, Scopus and EMBASE until December 2024. Pooled data were reported using risk ratio (RR) for dichotomous outcomes and mean difference (MD) for continuous outcomes, along with a 95% confidence interval (CI). This systematic review and meta‐analysis is registered with PROSPERO ID: CRD42024612005.

**Results:**

We included three RCTs with 885 patients. Pioglitazone add‐on therapy significantly reduced HbA1c levels (MD: −0.41; 95% CI: −0.54 to −0.27, *p* = < 0.00001, *I* ^2^ = 0%), fasting blood glucose (MD: −11.91; 95% CI: −16.34 to −7.48, *p* = < 0.00001, *I* ^2^ = 0%), Homeostatic Model Assessment for Insulin Resistance (HOMA‐IR) (MD: −0.65; 95% CI: −1.05 to −0.25, *p* = 0.001, *I* ^2^ = 4.89%), increased the rate of achieving HbA1c < 7% (RR: 2.09; 95% CI: 1.66 to 2.64, *p* = < 0.00001, *I* ^2^ = 0%), and HbA1c < 6.5% (RR: 2.19; 95% CI: 1.36 to 3.53, *p* = 0.001, *I* ^2^ = 0%). However, there was no difference regarding Homeostasis model assessment of β‐cell function (HOMA‐β) between the two groups (MD: 2.73; 95% CI: −5.24 to 10.70, *p* = 0.5, *I* ^2^ = 27.53%).

**Conclusion:**

Pioglitazone add‐on therapy significantly improved glycaemic control by reducing HbA1c, fasting blood glucose and HOMA‐IR while increasing the likelihood of achieving HbA1c targets. However, no significant difference was observed in HOMA‐β between groups. These findings suggest the potential benefit of pioglitazone in enhancing glycaemic outcomes in diabetes management.

## Graphical abstract

Pioglitazone add‐on to metformin and dapagliflozin significantly improved glycaemic control by reducing HbA1c, fasting blood glucose and HOMA‐IR while increasing the likelihood of achieving HbA1c targets in inadequately controlled type 2 diabetes, without compromising safety. This meta‐analysis highlights its potential as a third‐line therapy, despite associated weight gain and no impact on β‐cell function.

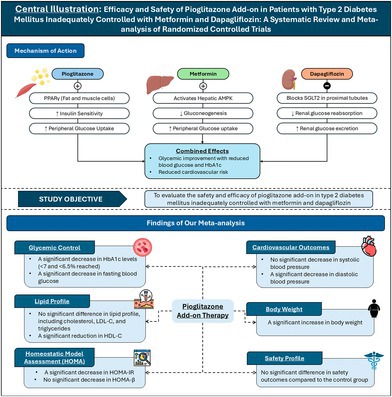

## 1 Introduction

Type 2 diabetes mellitus (T2DM) accounts for over 90% of diabetes cases worldwide [1]. Its pathophysiology primarily involves progressive dysfunction of pancreatic β‐cells and deteriorating insulin sensitivity in peripheral tissues [2]. Most patients with T2DM require a combination of oral antidiabetic drugs (OADs) with different mechanisms of action to achieve glycaemic control and prevent long‐term complications [3]. When two‐drug therapy fails to achieve euglycemia, a third agent is typically added to the regimen.

Sodium‐glucose cotransporter‐2 (SGLT2) inhibitors have become a standard add‐on option due to their unique insulin‐independent mechanism, which promotes urinary glucose excretion by inhibiting glucose reabsorption in the proximal renal tubule [4]. This mechanism minimises the risk of hypoglycaemia while conferring benefits on visceral adiposity, hyperuricaemia, lipid profile and blood pressure [5]. Multiple clinical trials have demonstrated that SGLT2 inhibitors improve cardiovascular and renal outcomes, primarily attributed to their osmotic diuretic effect [6, 7, 8, 9]. However, despite their favourable safety profile, they are associated with adverse events such as diabetic ketoacidosis (DKA), genitourinary infections and hypotension [8, 10]. Current guidelines by the American Diabetes Association (ADA), the European Association for the Study of Diabetes (EASD) and the American Association of Clinical Endocrinology endorse SGLT2 inhibitors as a core component of T2DM management, particularly for patients with atherosclerotic cardiovascular disease, heart failure, or chronic kidney disease [11, 12].

Pioglitazone, a thiazolidinedione, works by activating the nuclear hormone receptor peroxisome proliferator‐activated receptor‐γ (PPAR‐γ) [13], thereby enhancing insulin sensitivity in various tissues [14]. Extensive clinical trials have demonstrated that pioglitazone significantly reduces the risk of myocardial infarction and stroke [15, 16] and may also slow the progression of atherosclerosis [17, 18]. However, side effects, including weight gain and plasma volume expansion, which may precipitate heart failure, have limited its use as a monotherapy [19]. Combining pioglitazone with SGLT2 inhibitors may provide a synergistic effect by mitigating pioglitazone‐associated fluid retention and weight gain without increasing the risk of hypoglycaemia [20, 21].

Evidence on the efficacy and safety of adding pioglitazone as a third agent for patients with T2DM inadequately controlled on metformin and SGLT2 inhibitors is limited. This systematic review and meta‐analysis aims to evaluate the efficacy and safety of pioglitazone as an add‐on therapy for patients with T2DM who are not achieving glycaemic targets with dual therapy.

## 2 Methodology

### 2.1 Protocol Registration

This systematic review and meta‐analysis were completed using the PRISMA statement [22] and the Cochrane Handbook for systematic reviews and meta‐analyses [23]. This review has been registered in PROSPERO under the following ID: CRD42024612005.

### 2.2 Data Sources and Search Strategy

Our search was conducted until December 2024 on the following databases: PubMed (MEDLINE), Web of Science (WOS), SCOPUS, EMBASE and the Cochrane Central Register of Controlled Trials (CENTRAL). Following our modifications to each database's search terms and keywords, the detailed search strategy is shown in (Table S1).

### 2.3 Eligibility Criteria

We included randomised controlled trials (RCTs) that followed the following PICO criteria: population (P): patients with T2DM; intervention (I): pioglitazone add‐on therapy with metformin and dapagliflozin; comparison (C): metformin and dapagliflozin; and outcomes (O): our primary outcomes are the change in HbA1c, achievement of HbA1c targets of < 7% and < 6.5%. Our secondary outcomes included the change in fasting blood glucose, HOMA‐IR, HOMA‐â, systolic blood pressure (SBP), diastolic blood pressure (DBP), total cholesterol, Low‐density lipoprotein‐cholesterol (LDL‐C), High‐density lipoprotein‐cholesterol (HDL‐C), triglycerides (TAGs), body weight and safety outcomes, including treatment‐emergent adverse events (TEAE) and adverse drug reactions (ADR). The following criteria were used to exclude papers: the following study types are not considered original: (1) book chapters, reviews, comments, letters to the editor and guidelines; (2) any other study design other than RCTs; (3) studies with overlapping or duplicate datasets; (4) non‐human and in vitro experiments; and (5) studies not published in English.

### 2.4 Study Selection

The review was carried out using the Covidence online tool. After eliminating duplicates, two authors (U.K. and M.H.) evaluated each obtained record independently. During the full‐text screening for eligibility criteria, two authors (A.M.A. and M.H.) reviewed the full texts of the documents. Any differences were settled through discussion and agreement with the first author.

### 2.5 Data Extraction

To set up the data extraction sheet accurately, we performed a pilot extraction after obtaining the full texts of the relevant publications. The Excel (Microsoft, U.S.A.) structured data extraction sheet is divided into three sections. The first part included the summary characteristics of the included studies (study ID, country, study design, number of centers, registry number, blinding status, inclusion criteria of the RCT), intervention group, control (comparison group), sample size, primary outcome and follow‐up period. The second part included the baseline information of the participants (number of patients in each group, age, gender, SBP, DBP, HbA1c, heart rate, Estimated Glomerular Filtration Rate (eGFR), smoking, baseline medications, other anti‐hyperglycaemic medications, non‐ST‐elevation myocardial infarction (NSTEMI), ST‐elevation myocardial infarction (STEMI), angina and comorbidities). Finally, the third part included outcomes data. Two reviewers (A.M.A. and Z.M.) were responsible for data extraction. Any differences were settled by discussion and agreement with a senior author.

### 2.6 Risk of Bias and Certainty of Evidence

Two reviewers (Z.M. and A.M.A.) independently evaluated the studies' quality using the Cochrane ROB‐2 method [24]. They considered five domains, including the risk of bias associated with the randomisation process, deviation from the intended intervention, missing outcome data, measuring the outcome and choosing the reported results. Any disagreements were resolved by discussion with a senior author.

### 2.7 Statistical Analysis

The statistical analysis was conducted using RevMan v5.3 software. For dichotomous outcomes, we employed the risk ratio (RR), while for continuous outcomes, we utilised the mean difference (MD), both reported with a 95% confidence interval (CI). To evaluate heterogeneity, we used the chi‐square and *I* ^2^ tests. The chi‐square test was employed to identify the presence of heterogeneity, while the *I* ^2^ test gauged its degree. Our interpretation of the *I* ^2^ test followed these criteria: heterogeneity is insignificant for 0%–40%, moderate for 30%–60%, substantial for 50%–90% and considerable for 75%–100%, as outlined in the Cochrane Handbook (chapter nine). We considered a significance level below 0.1 for the chi‐square test to indicate significant heterogeneity.

## 3 Results

### 3.1 Search Results and Study Selection

**FIGURE 1:**
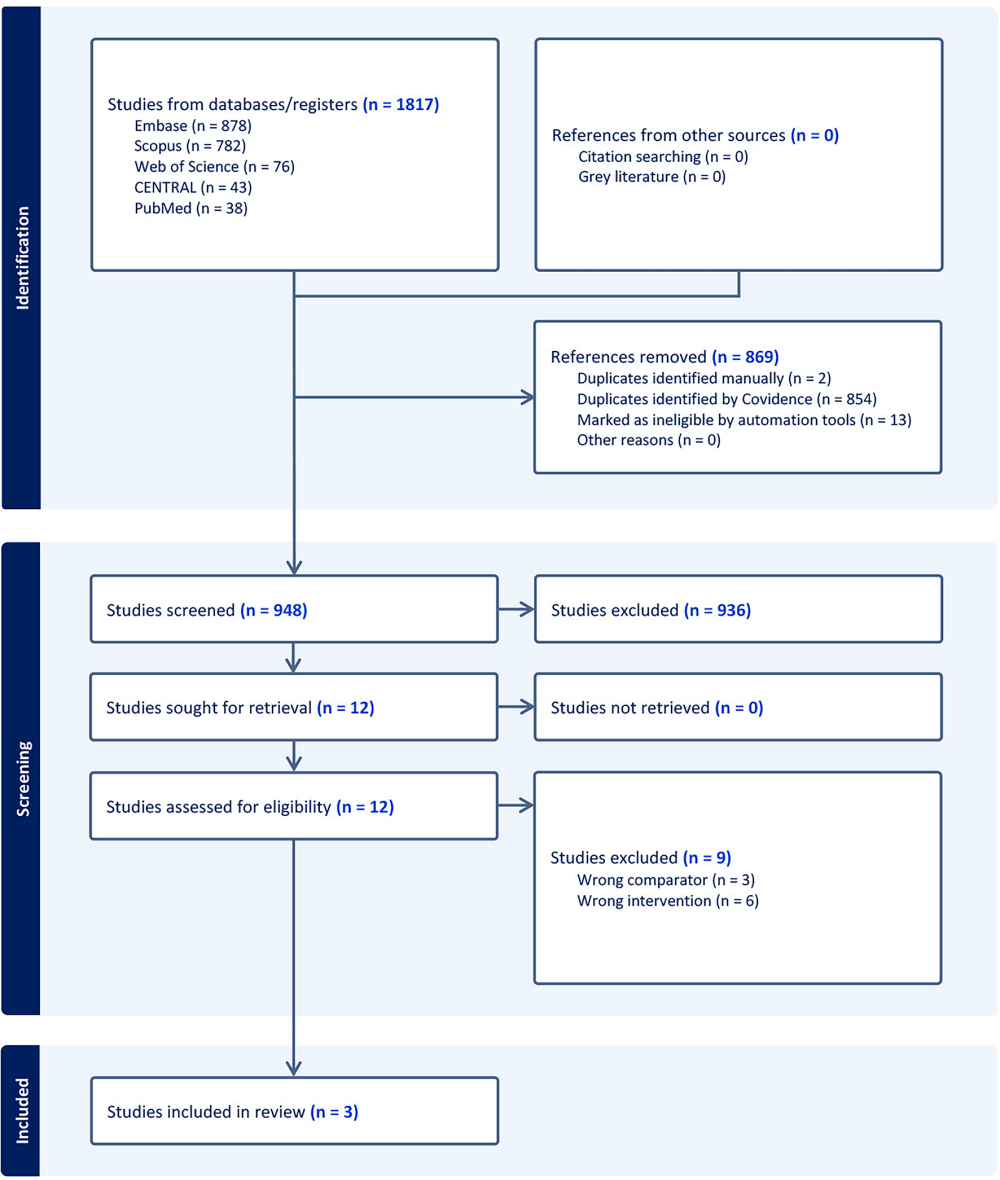
PRISMA flow chart of the screening process.

A total of 1817 records were incorporated from five databases into Covidence. Eight hundred sixty‐nine were duplicates and removed by Covidence, leaving 948 records to be screened. Out of these, 936 records were found to be irrelevant and excluded in title and abstract screening. This left 12 studies for full‐text screening, and three were found eligible for data extraction (Figure 1).

### 3.2 Characteristics of Included Studies

The final analysis included three RCTs [25, 26, 27] with 885 patients. The mean age of patients ranged from 56.8 to 57.7 years, while the mean HbA1c of patients ranged from 7.62% to 7.92%. The majority of participants were male. Comprehensive details of the included studies' summary characteristics and the participants' baseline characteristics are outlined in (Tables 1 and 2).

**TABLE 1:** Study characteristics of included trials.

Study	Study design	Country	Total participants	Intervention	Control	Main inclusion criteria	Follow up duration	Primary outcome
Lim et al. (2024) [25]	Double‐blind, RCT	South Korea	249	Pioglitazone (15 mg daily), Metformin (1383 + _ 478 mg), dapagliflozin (10 mg)	Placebo, metformin (1402 + _ 413), dapagliflozin (10 mg)	Participants 19 years or older, T2DM, body mass index (BMI) ≤ 40.0 kg/m^2^ at screening, receiving metformin ≥ 1000 mg/day plus dapagliflozin 10 mg/day dual therapy for 8 weeks or more before screening, and baseline HbA1c 7.0%–10.5%.	48 weeks	Changes in HbA1c at 24 weeks from baseline
Heo et al. (2024) [26]	Multi‐center, Double‐blind, RCT	South Korea	262	Pioglitazone (15 mg daily), dapagliflozin (10 mg/day), metformin (≥ 1000 mg/day).	Placebo, dapagliflozin (10 mg/day), metformin (≥ 1000 mg/day).	Patients ≥ 19 years of age with T2DM and a body mass index ≤ 45 kg/m^2^, receiving metformin ≥ 1000 mg/day plus dapagliflozin 10 mg/day, HbA1c of 7%–11% at screening.	34 weeks	Changes in HbA1c at 24 weeks from baseline
Cho et al. (2024) [27]	Multi‐center double‐blinded, phase III RCT	South Korea	374	Pioglitazone (15 mg daily) or Pioglitazone (30 mg daily), dapagliflozin (10 mg/day), metformin (≥ 1000 mg/day).	Placebo, dapagliflozin (10 mg/day), metformin (≥ 1000 mg/day).	Patients ≥ 19 years of age with T2DM, receiving metformin ≥ 1000 mg/day plus dapagliflozin 10 mg/day for at least 8 weeks, HbA1c of 7%–10.5% at screening.	48 weeks	Changes in HbA1c at 24 weeks from baseline

Abbreviations: HbAic, haemoglobin A1C; Mg, milligram; RCT, randomised controlled trial; T2DM, type 2 diabetes mellitus.

**TABLE 2:** Baseline details of included trials.

Study ID	Number of patients in each group		Age (years), mean (SD)		Gender (Male), *N* (%)		SBP, mean (SD)		DBP, mean (SD)		HbA1c (%)		Heart rate, mean (SD)		eGFR (mL/min/1.73 m^2^), mean (SD)		Smoking, mean (SD)		HTN	
	Pioglitazone add‐on	Control	Pioglitazone add‐on	Control	Pioglitazone add‐on	Control	Pioglitazone add‐on	Control	Pioglitazone add‐on	Control	Pioglitazone add‐on	Control	Pioglitazone add‐on	Control	Pioglitazone add‐on	Control	Pioglitazone add‐on	Control	Pioglitazone add‐on	Control
Lim et al. (2024) [25]	124	125	57.7 (10.0)	58.2 (10.1)	70 (56.5)	64 (51.2)	124.8 (13.8)	125.2 (14.3)	74.3 (9.5)	74.3 (9.5)	7.8 (0.7)	7.8 (0.8)	76 (9.2)	77.4 (10.8)	92.3 (15.9)	91.1 (13.7)	27 (21.8)	21 (16.8)	62 (50)	74 (59.2)
Heo et al. (2024) [26]	131	131	57.6 (10.0)	56.9 (10.3)	61 (48.8)	60 (48.0)	127.32 (11.57)	127.37 (11.92)	76.41 (9.02)	77.34 (8.51)	7.62 (0.55)	7.76 (0.77)	NA	NA	87.98 (19.11)	92.53 (20.15)	18 (14.4)	29 (23.2)	NA	NA
Cho et al. (2024) [27]	118	124	56.8 (11)	55 (10.3)	73 (61.9)	66 (53.2)	123.6 (12.1)	126 (13.4)	75.1 (9.4)	75.9 (9.4)	7.9 (0.7)	7.9 (0.7)	NA	NA	95 (15)	95.5 (14.9)	NA	NA	53 (44.9)	67 (54)

Abbreviations: DBP, diastolic blood pressure; eGFR, estimated glomerular filtration rate; HbAic, haemoglobin A1C; HTN, hypertension; SBP, systolic blood pressure.

### 3.3 Risk of Bias and Certainty of Evidence

**FIGURE 2:**
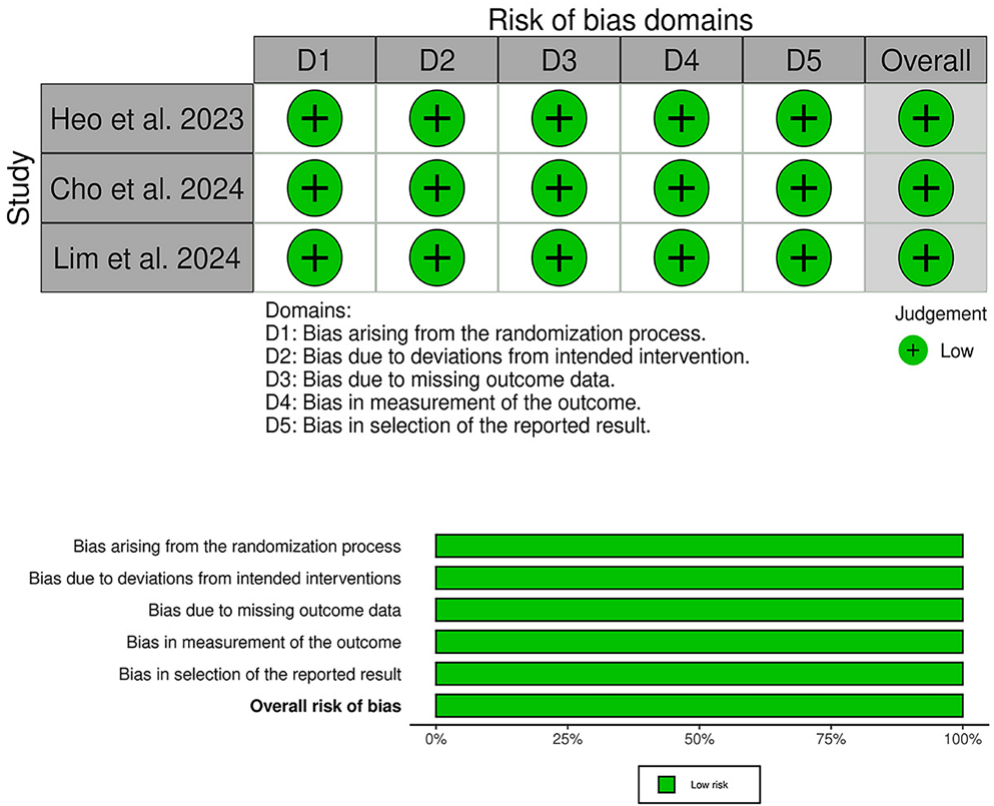
Quality assessment of risk of bias in the included trials. The upper panel presents a schematic representation of risks (low = green, unclear = yellow and high = red) for specific types of biases of each study in the review. The lower panel presents risks (low = green, unclear = yellow and high = red) for the subtypes of biases of the combination of studies included in this review.

After assessing three RCTs by ROB‐2, all RCTs included in the analysis demonstrated a low risk of bias, as comprehensively outlined in (Figure 2). Certainty of evidence is demonstrated in the GRADE evidence profile (Table 3). The Rob2 details for each domain assessed are explained in (Tables S2–S4).

**TABLE 3:** GRADE evidence profile.

Certainty assessment							Summary of findings				
Participants (studies) follow‐up	Risk of bias	Inconsistency	Indirectness	Imprecision	Publication bias	Overall certainty of evidence	Study event rates (%)		Relative effect (95% CI)	Anticipated absolute effects	
							With [control]	With [pioglitazone add‐on]		Risk with [control]	Risk difference with [pioglitazone add‐on]
*HbA1c change*											
732 (3 RCTs)	Not serious	Not serious	Not serious	Serious^a^	None	⨁⨁⨁◯ Moderate^a^	369	363	—	369	MD 0.41% lower (0.54 lower to 0.27 lower)
*HbA1c target of < 7%*											
716 (3 RCTs)	Not serious	Not serious	Not serious	Serious^b^	None	⨁⨁⨁◯ Moderate^b^	74/361 (20.5%)	153/355 (43.1%)	RR 2.09 (1.66 to 2.64)	74/361 (20.5%)	223 more per 1000 (from 135 more to 336 more)
*HbA1c target of < 6.5%*											
718 (3 RCTs)	Not serious	Not serious	Not serious	Serious^b^	None	⨁⨁⨁◯ Moderate^b^	22/361 (6.1%)	48/357 (13.4%)	RR 2.19 (1.36 to 3.53)	22/361 (6.1%)	73 more per 1000 (from 22 more to 154 more)
*Fasting blood glucose change*											
732 (3 RCTs)	Not serious	Not serious	Not serious	Serious^a^	None	⨁⨁⨁◯ Moderate^a^	369	363	—	369	MD 11.91 mg/dL lower (16.34 lower to 7.48 lower)
*HOMA‐IR change*											
732 (3 RCTs)	Not serious	Not serious	Not serious	Serious^a^	None	⨁⨁⨁◯ Moderate^a^	369	363	—	369	MD 0.65 lower (1.05 lower to 0.25 lower)
*Treatment‐emergent adverse events*											
757 (3 RCTs)	Not serious	Not serious	Not serious	Very serious^c^	None	⨁⨁◯◯ Low^c^	98/380 (25.8%)	101/377 (26.8%)	RR 1.04 (0.82 to 1.32)	98/380 (25.8%)	10 more per 1000 (from 46 fewer to 83 more)
*Adverse drug reactions*											
757 (3 RCTs)	Not serious	Not serious	Not serious	Very serious^c^	None	⨁⨁◯◯ Low^c^	9/380 (2.4%)	19/377 (5.0%)	RR 2.14 (0.99 to 4.66)	9/380 (2.4%)	27 more per 1000 (from 0 fewer to 87 more)

Abbreviations: CI, confidence interval; MD, mean difference; RR, risk ratio.

^a^ A wide confidence interval that does not exclude the appreciable harm or benefit with a low number of patients included in the pooled analysis.

^b^ Low number of events (< 300 events).

^c^ A wide confidence interval that does not exclude the appreciable harm or benefit with a low number of events included in the pooled analysis.

### 3.4 Primary Outcomes

**FIGURE 3:**
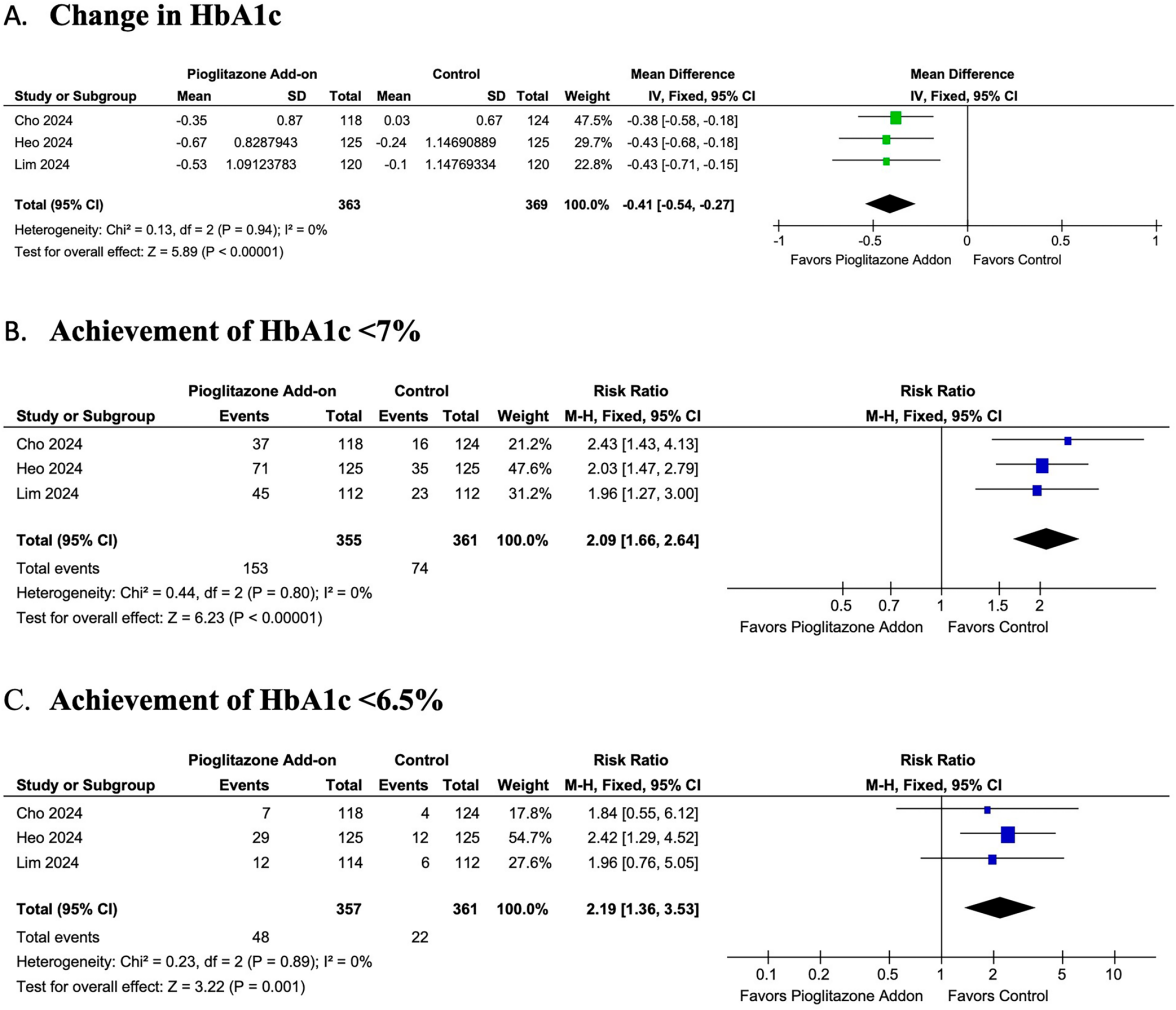
Forest plot of the (A) change in HbA1c levels, (B) achievement of HbA1c < 7% and (C) achievement of HbA1c < 6.5%. CI, confidence interval; RR: risk ratio; SD, standard deviation.

Pioglitazone as an add‐on therapy demonstrated significant efficacy in improving glycaemic control, with a significant reduction in HbA1c levels compared to the control group (MD: −0.41; 95% CI: −0.54 to −0.27, *p* = < 0.00001, *I* ^2^ = 0%) (Figure 3A). Additionally, a significantly higher proportion of patients achieved the target HbA1c levels of < 7% (RR: 2.09; 95% CI: 1.66 to 2.64, *p* = < 0.00001, *I* ^2^ = 0%) (Figure 3B) and HbA1c < 6.5% (RR: 2.19; 95% CI: 1.36 to 3.53, *p* = 0.001, *I* ^2^ = 0%) (Figure 3C).

### 3.5 Secondary Outcomes

#### 3.5.1 Endocrine Outcomes

**FIGURE 4:**
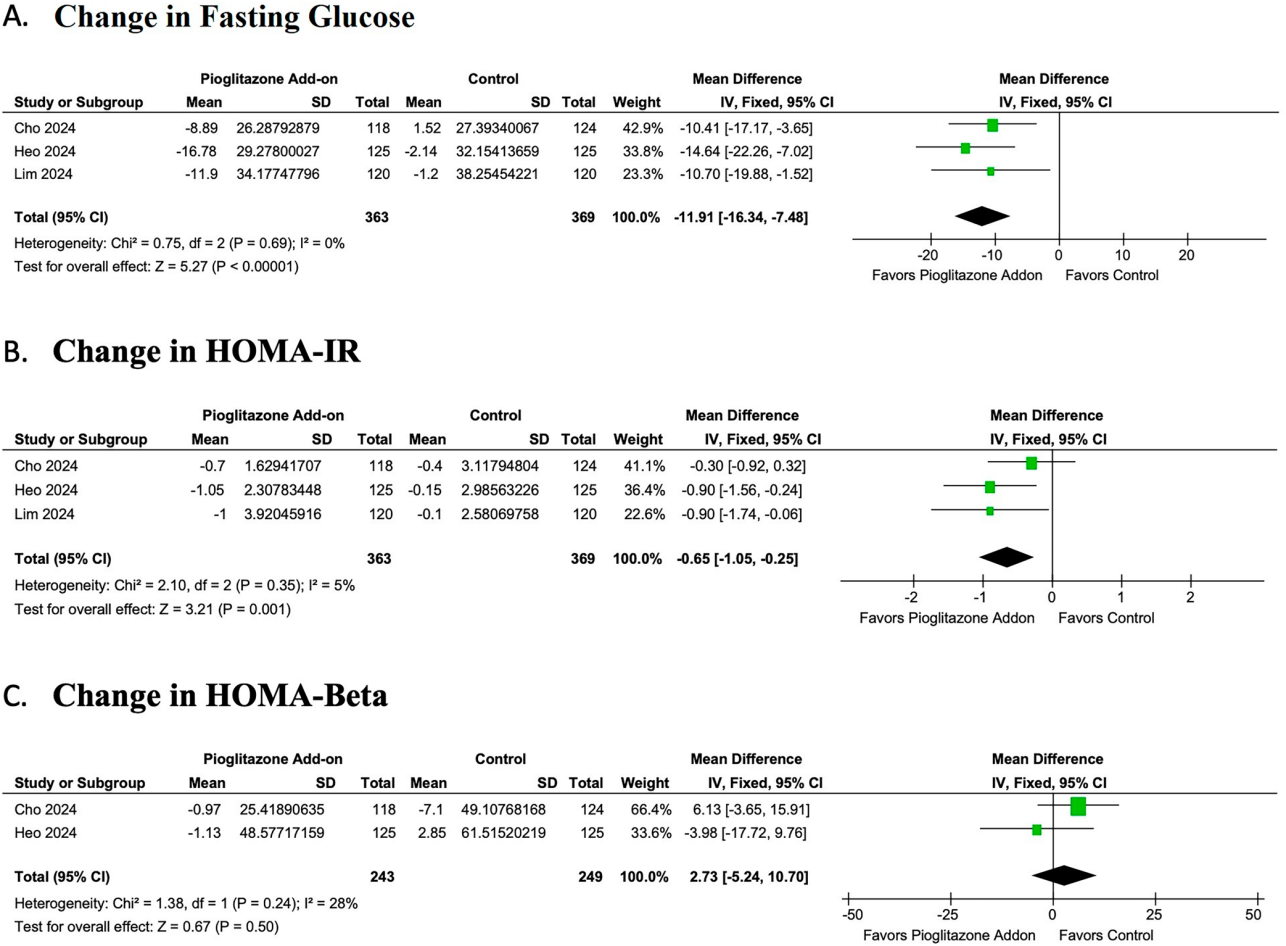
Forest plots of the (A) change in fasting blood glucose, (B) HOMA‐IR and (C) HOMA‐Beta. CI, confidence interval; SD, standard deviation.

Pioglitazone add‐on therapy showed a significant reduction in fasting blood glucose levels (MD: −11.91; 95% CI: −16.34 to −7.48, *p* = < 0.00001, *I* ^2^ = 0%) (Figure 4A) and a significant reduction in HOMA‐IR (MD: −0.65; 95% CI: −1.05 to −0.25, *p* = 0.001, *I* ^2^ = 4.89%) (Figure 4B). However, no statistically significant difference was noted in HOMA‐β between the pioglitazone and control groups (MD: 2.73; 95% CI: −5.24 to 10.70, *p* = 0.5, *I* ^2^ = 27.53%) (Figure 4C).

#### 3.5.2 Cardiovascular Outcomes

**FIGURE 5:**
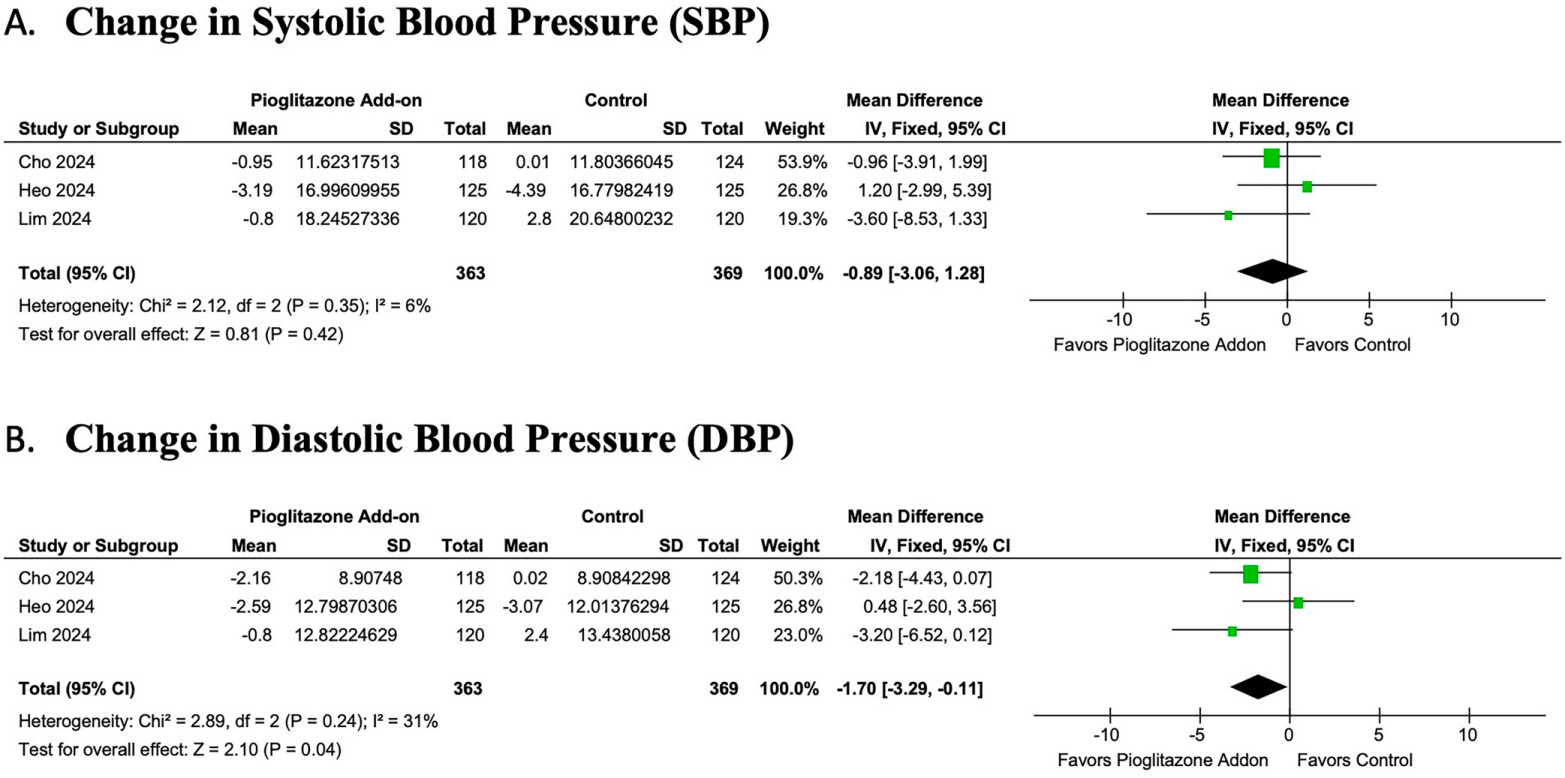
Forest plots of the (A) change in systolic blood pressure and (B) change in diastolic blood pressure. CI, confidence interval; SD, standard deviation.

Pioglitazone add‐on therapy had varying effects on cardiovascular outcomes. No statistically significant change was observed in SBP between the two groups (MD: −0.89; 95% CI: −3.06 to 1.28, *p* = 0.42, *I* ^2^ = 5.67%) (Figure 5A). However, a statistically significant reduction was noted in DBP (MD: −1.70; 95% CI: −3.29 to −0.11, *p* = 0.04, *I* ^2^ = 30.7%) (Figure 5B).

#### 3.5.3 Lipid Profile

**FIGURE 6:**
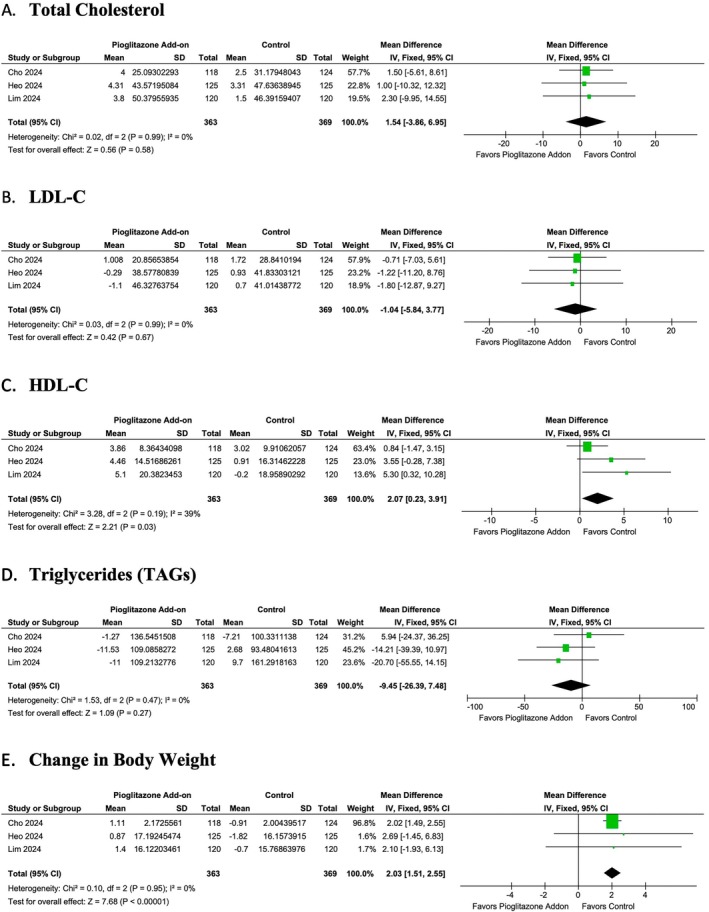
Forest plot of the (A) total cholesterol, (B) LDL‐C, (C) HDL‐C, (D) triglycerides and (E) change in body weight. CI, confidence interval; SD, standard deviation.

Pioglitazone add‐on therapy demonstrated mixed effects on the lipid profile. No statistically significant impact was observed on total cholesterol (MD: 1.54; 95% CI: −3.86 to 6.95, *p* = 0.58, *I* ^2^ = 0%) (Figure 6A), LDL‐C (MD: −1.04; 95% CI: −5.84 to 3.77, *p* = 0.67, *I* ^2^ = 0%) (Figure 6B) and triglycerides (MD: −9.45; 95% CI: −26.39 to 7.48, *p* = 0.27, *I* ^2^ = 0%) (Figure 6C). However, a statistically significant increase was observed in HDL‐C levels with add‐on therapy (MD: 2.07; 95% CI: 0.23 to 3.91, *p* = 0.03, *I* ^2^ = 39.06%) (Figure 6D). Additionally, pioglitazone add‐on therapy led to a significant increase in body weight (MD: 2.03; 95% CI: 1.51 to 2.55, *p* < 0.00001, *I* ^2^ = 0%) (Figure 6E).

#### 3.5.4 Safety Outcomes

**FIGURE 7:**
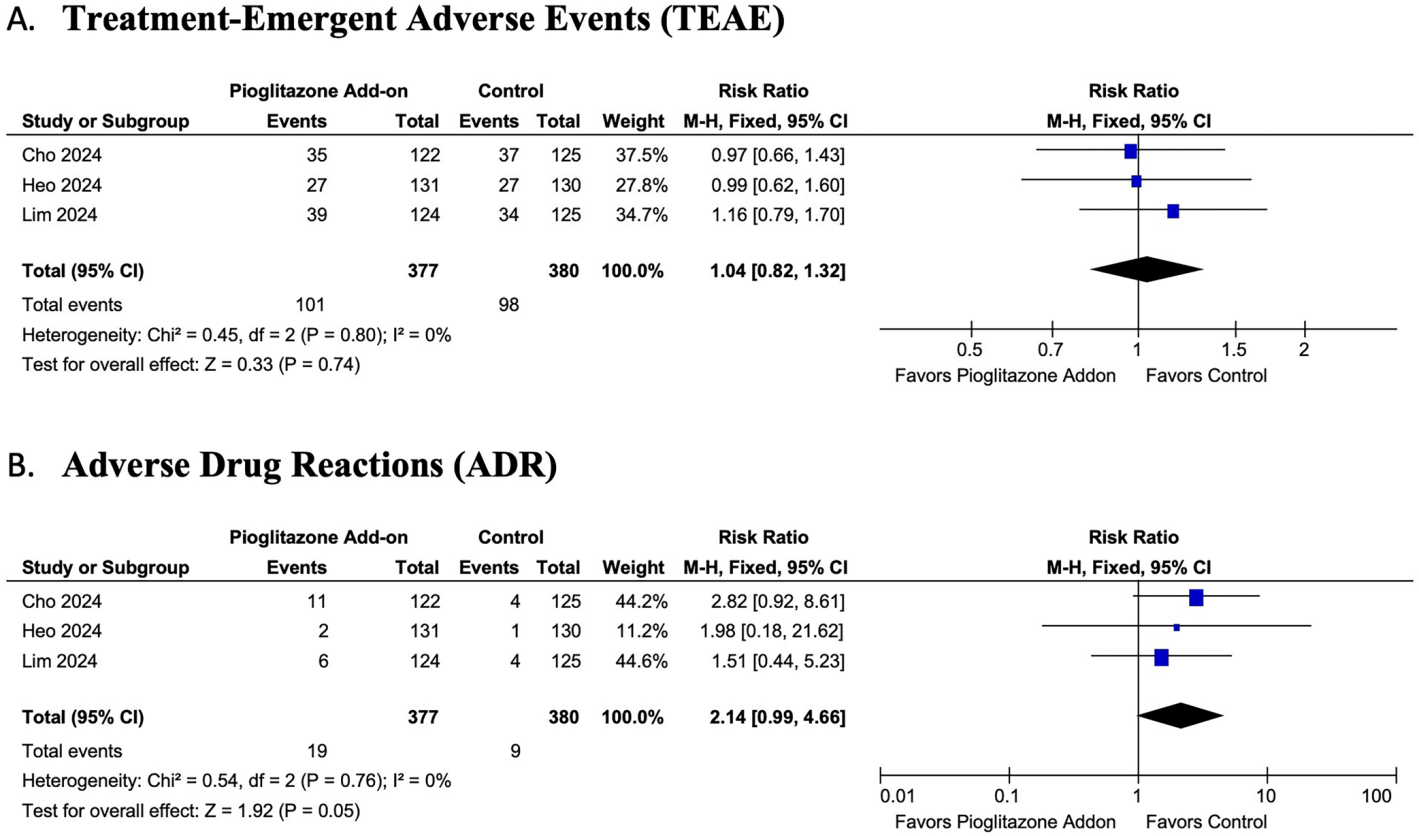
Forest plot of the (A) treatment emergent adverse events and (B) adverse drug reaction. CI, confidence interval; RR, risk ratio.

Pioglitazone add‐on therapy showed no statistically significant differences in safety outcomes compared to the control group. There was no significant difference in TEAE (RR: 1.04; 95% CI: 0.82 to 1.32, *p* = 0.74, *I* ^2^ = 0%) (Figure 7A) and ADR (RR: 2.14; 95% CI: 0.99 to 4.66, *p* = 0.05, *I* ^2^ = 0%) (Figure 7B).

## 4 Discussion

The primary findings demonstrated a significant reduction in HbA1c with pioglitazone add‐on therapy, accompanied by substantially higher achievement rates of both HbA1c < 7% and < 6.5% targets. These improvements in glycaemic control were consistent across studies, as evidenced by the negligible heterogeneity in our analyses. The magnitude of HbA1c reduction likely reflects pioglitazone's complementary mechanism of action, enhancing insulin sensitivity through PPAR‐γ activation [28], while working synergistically with the glucose excretion effects of SGLT2 inhibitors and metformin's reduction of hepatic glucose production [29, 30].

Secondary outcomes revealed meaningful improvements in several metabolic parameters, including fasting blood glucose and insulin resistance (HOMA‐IR), supporting pioglitazone's insulin‐sensitising effects [31]. As measured by HOMA‐β, beta‐cell function remained unchanged, suggesting that pioglitazone's primary mechanism operates through insulin sensitisation rather than beta‐cell preservation [32].

The lipid profile remained largely stable, with no significant alterations in total cholesterol, LDL‐C, or triglycerides. The observed increase in HDL‐C aligns with pioglitazone's known beneficial effects on lipid metabolism [33]. Notably, adding pioglitazone did not significantly impact most cardiovascular parameters, except for a modest reduction in diastolic blood pressure. The neutral effect on these parameters is particularly noteworthy as it suggests that pioglitazone addition does not adversely affect the cardiovascular and metabolic benefits of SGLT2 inhibitor therapy [29]. However, the anticipated side effect of weight gain (MD: 2.03 kg) was confirmed, though this may have been partially attenuated by the concurrent use of SGLT2 inhibitors [34]. The complementary cardiovascular mechanisms of these agents merit consideration. SGLT2 inhibitors primarily reduce heart failure hospitalisation through hemodynamic effects [35], while pioglitazone's cardiovascular benefits appear mediated through impacts on coronary atherosclerosis progression as demonstrated in the PERISCOPE trial [17] and suggested in the PROactive study [36]. This mechanistic complementarity provides a theoretical basis for additive cardiovascular protection, though longer‐duration studies are needed to confirm combined effects.

The safety profile was particularly encouraging, with no significant increase in treatment‐emergent adverse events or adverse drug reactions, indicating that the triple therapy combination was generally well‐tolerated. This favourable safety profile may be attributed to the moderate pioglitazone dosing (15 mg daily) used in the included studies and the potential offsetting of fluid retention by SGLT2 inhibitors' natriuretic effects.

While the safety profile of pioglitazone in combination therapy is generally favourable, it may not fully capture adverse events such as fluid retention, which can worsen heart failure in susceptible individuals [37]. Although SGLT2 inhibitors might mitigate this risk, the studies did not report on peripheral edema or heart failure events. Additionally, the increased risk of bone fractures, especially in postmenopausal women, was not adequately addressed due to the short study duration [38, 39].

Our findings both complement and extend previous meta‐analyses examining combination therapies in T2DM. The landmark meta‐analysis by Zhang et al. compared the efficacy and safety of SGLT2 inhibitors and metformin in adults with DM. SGLT2 inhibitors significantly reduced HbA1c levels and body weight compared to placebo and metformin. SGLT2 inhibitors and metformin reduced total insulin dosage compared to placebo, with no significant difference. There was no difference in the risk of severe hypoglycaemia between SGLT2 inhibitors and metformin. However, SGLT2 inhibitors carried a higher risk of DKA than metformin or placebo. Overall, SGLT2 inhibitors were more effective but posed a higher DKA risk. Examining dual therapy with SGLT2 inhibitors and metformin reported an HbA1c reduction of −0.40% [40].

Notably, a comprehensive network meta‐analysis by Downes et al. evaluated triple therapy regimens available in Australia, analysing evidence from 27 trials conducted between 2002 and 2014 [41]. Their findings demonstrated that virtually all triple therapy combinations, except metformin‐thiazolidinedione‐DPP4 inhibitor combinations, achieved superior glycaemic control compared to dual therapy with metformin and sulfonylureas. The variable outcomes regarding weight changes and hypoglycaemia risk across different combinations in their analysis underscore the importance of individualised therapy selection, particularly given our observed weight gain with pioglitazone addition.

The findings of our meta‐analysis have several important clinical implications. First, they establish pioglitazone as an effective and safe third‐line option for patients not achieving glycaemic targets on metformin and SGLT2 inhibitors, particularly when cost considerations preclude using GLP‐1 receptor agonists [42]. The consistent glycaemic improvements across studies suggest that this combination could be especially valuable for patients with marked insulin resistance.

When considering pioglitazone as a third‐line agent, it is important to compare its efficacy and safety with alternatives like GLP‐1 receptor agonists (GLP‐1RAs) and DPP‐4 inhibitors. GLP‐1RAs offer strong glycaemic control, weight loss and cardiovascular benefits but may be limited by injection requirements and cost. DPP‐4 inhibitors are an oral option with low hypoglycaemia risk but provide modest HbA1c reductions [43]. The choice should depend on factors such as cardiovascular risk, weight concerns, hypoglycaemia risk, cost and patient preferences. Pioglitazone may benefit patients with high insulin resistance, while GLP‐1RAs may be better for those with obesity [44]. This personalised approach aligns with patient‐centred care guidelines.

While significant, the modest weight gain observed with pioglitazone addition appears less pronounced than historically reported with pioglitazone monotherapy, suggesting a partial counterbalancing effect of SGLT2 inhibitors [34]. This finding may help clinicians in shared decision‐making discussions with patients, particularly when weighing the benefits of improved glycaemic control against potential weight effects.

The neutral cardiovascular safety profile and improved HDL‐C levels suggest that this triple therapy combination may suit patients with established cardiovascular disease. However, more extensive outcome studies would be needed to confirm cardiovascular benefits [45].

## 5 Strength and Limitations

Our meta‐analysis provides the most comprehensive evidence supporting the efficacy of pioglitazone as a third‐line agent in patients with T2DM inadequately controlled on metformin and SGLT2 inhibitors. Further strengths are the exclusive inclusion of RCTs with a low risk of bias across all domains and the consistency of findings across studies, as evidenced by low heterogeneity for most outcomes, enhancing our conclusions' reliability. The comprehensive assessment of efficacy and safety outcomes provides clinicians with a complete picture for informed decision‐making. Additionally, the uniform dosing of medications across studies (pioglitazone 15 mg, dapagliflozin 10 mg) and standardised metformin background therapy facilitate clear interpretation of the results.

However, several limitations should be acknowledged. A key limitation is the inclusion of only three RCTs, which, although high‐quality, may not capture the full spectrum of treatment effects and could make our findings more susceptible to the small‐study impacts. All included studies were conducted in South Korea, potentially limiting generalisability to other populations with different genetic backgrounds, dietary habits and healthcare systems. The relatively short follow‐up duration (24–48 weeks) precludes long‐term outcomes and rare adverse events assessment. Also, the lack of active comparator arms prevents direct comparison with other third‐line agents, such as GLP‐1 receptor agonists or DPP‐4 inhibitors. Finally, the absence of patient‐reported outcomes and quality‐of‐life measures restricts our understanding of the treatment's impact on patient experience.

## 6 Implications for Future Research

Future research should focus on longer‐term cardiovascular outcomes, optimal dosing strategies and effectiveness in diverse populations. Additionally, head‐to‐head comparisons with other third‐line agents would help further define pioglitazone's place in the treatment algorithm. Future studies need to include a large number of patients to increase the power of the findings.

## 7 Conclusion

This meta‐analysis provides strong evidence supporting the addition of pioglitazone to metformin and SGLT2 inhibitor therapy in patients with inadequately controlled T2DM. The combination demonstrates significant glycaemic benefits with an acceptable safety profile, though careful weight monitoring is warranted. Based on these findings, we recommend considering pioglitazone as a cost‐effective third‐line option, particularly in patients with features of insulin resistance.

## Author Contributions

U.K.: conceived the idea. U.K.: designed the research workflow. M.H.K. and H.M.W.S.: searched the databases. A.M.A., M.H.K. and Z.M.: screened the retrieved records, extracted relevant data and assessed the quality of evidence. A.N.: resolved the conflicts. A.M.A.: performed the analysis. A.M.A., A.B.S. and M.A.: wrote the final manuscript. U.K. and M.T.: supervised the project. All authors have read and agreed to the final version of the manuscript.

## Ethics Statement

The authors have nothing to report.

## Consent

The authors have nothing to report.

## Conflicts of Interest

The authors declare no conflicts of interest.

## Supporting information


Data S1:

## Data Availability

The data is available upon reasonable request from the corresponding author.
