# Supplementary material for: Efficacy and Safety of Pioglitazone Add‐On in Patients With Type 2 Diabetes Mellitus Inadequately Controlled With Metformin and Dapagliflozin: A Systematic Review and Meta‐Analysis of Randomised Controlled Trials
Source: Endocrinol Diabetes Metab. 2025 May 27;8(3):e70061. doi: 10.1002/edm2.70061 (PMC12106948; doi:10.1002/edm2.70061)
Supplement: Supplementary file 1 — Data S1: [file EDM2-8-e70061-s001.docx]

**Supplementary Appendix.**

**Tables.**

**Table S1:** Data source and search strategy.

**Table S2**-**S4:** Detailed risk of bias assessment for each trial.

| Database | Search Strategy | Search field | Search Results |
| --- | --- | --- | --- |
| PUBMED | (Pioglitazone OR Pioglitazone Hydrochloride) AND (Metformin OR Dimethylbiguanidine OR Dimethylguanylguanidine OR Metformin Hydrochloride) AND (dapagliflozin) AND (Diabetes OR diabetes mellitus OR DM OR Type 2 Diabetes OR Type 2 Diabetes Mellitus OR DM2) | All fields | 38 |
| CENTRAL | (Pioglitazone OR Pioglitazone Hydrochloride) AND (Metformin OR Dimethylbiguanidine OR Dimethylguanylguanidine OR Metformin Hydrochloride) AND (dapagliflozin) AND (Diabetes OR diabetes mellitus OR DM OR Type 2 Diabetes OR Type 2 Diabetes Mellitus OR DM2) | All fields | 43 |
| WoS | (Pioglitazone OR Pioglitazone Hydrochloride) AND (Metformin OR Dimethylbiguanidine OR Dimethylguanylguanidine OR Metformin Hydrochloride) AND (dapagliflozin) AND (Diabetes OR diabetes mellitus OR DM OR Type 2 Diabetes OR Type 2 Diabetes Mellitus OR DM2) | All fields | 76 |
| Scopus | (Pioglitazone OR Pioglitazone Hydrochloride) AND (Metformin OR Dimethylbiguanidine OR Dimethylguanylguanidine OR Metformin Hydrochloride) AND (dapagliflozin) AND (Diabetes OR diabetes mellitus OR DM OR Type 2 Diabetes OR Type 2 Diabetes Mellitus OR DM2) | All fields | 782 |
| EMBASE | (Pioglitazone OR Pioglitazone Hydrochloride) AND (Metformin OR Dimethylbiguanidine OR Dimethylguanylguanidine OR Metformin Hydrochloride) AND (dapagliflozin) AND (Diabetes OR diabetes mellitus OR DM OR Type 2 Diabetes OR Type 2 Diabetes Mellitus OR DM2) | All fields | 878 |

**Table S1** showing detailed Seacrch strategy for all the databases searched

| **Domain** | **Signalling question** | **Response** | **Comments** |
| --- | --- | --- | --- |
| **Bias arising from the randomization process** | 1.1 Was the allocation sequence random? | Y |  |
|  | 1.2 Was the allocation sequence concealed until participants were enrolled and assigned to interventions? | PY |  |
|  | 1.3 Did baseline differences between intervention groups suggest a problem with the randomization process? | N |  |
|  | **Risk of bias judgment** | **Low** |  |
| **Bias due to deviations from intended interventions** | 2.1. Were participants aware of their assigned intervention during the trial? | N |  |
|  | 2.2. Were carers and people delivering the interventions aware of participants' assigned intervention during the trial? | N |  |
|  | 2.3. If Y/PY/NI to 2.1 or 2.2: Were there deviations from the intended intervention that arose because of the experimental context? | N/A |  |
|  | 2.4 If Y/PY to 2.3: Were these deviations likely to have affected the outcome? | N/A |  |
|  | 2.5. If Y/PY/NI to 2.4: Were these deviations from intended intervention balanced between groups? | N/A |  |
|  | 2.6 Was an appropriate analysis used to estimate the effect of assignment to intervention? | Y |  |
|  | 2.7 If N/PN/NI to 2.6: Was there potential for a substantial impact (on the result) of the failure to analyse participants in the group to which they were randomized? | N/A |  |
|  | **Risk of bias judgment** | **Low** |  |
| **Bias due to missing outcome data** | 3.1 Were data for this outcome available for all, or nearly all, participants randomized? | Y |  |
|  | 3.2 If N/PN/NI to 3.1: Is there evidence that result was not biased by missing outcome data? | N/A |  |
|  | 3.3 If N/PN to 3.2: Could missingness in the outcome depend on its true value? | N/A |  |
|  | 3.4 If Y/PY/NI to 3.3: Is it likely that missingness in the outcome depended on its true value? | N/A |  |
|  | **Risk of bias judgment** | **Low** |  |
| **Bias in measurement of the outcome** | 4.1 Was the method of measuring the outcome inappropriate? | N |  |
|  | 4.2 Could measurement or ascertainment of the outcome have differed between intervention groups? | N |  |
|  | 4.3 Were outcome assessors aware of the intervention received by study participants? | PY |  |
|  | 4.4 If Y/PY/NI to 4.3: Could assessment of the outcome have been influenced by knowledge of intervention received? | PN |  |
|  | 4.5 If Y/PY/NI to 4.4: Is it likely that assessment of the outcome was influenced by knowledge of intervention received? | N/A |  |
|  | **Risk of bias judgment** | **Low** |  |
| **Bias in selection of the reported result** | 5.1 Were the data that produced this result analysed in accordance with a pre-specified analysis plan that was finalized before unblinded outcome data were available for analysis? | Y |  |
|  | 5.2 ... multiple eligible outcome measurements (e.g. scales, definitions, time points) within the outcome domain? | PN |  |
|  | 5.3 ... multiple eligible analyses of the data? | PN |  |
|  | **Risk of bias judgment** | **Low risk** |  |
| **Overall bias** | **Risk of bias judgment** | **Low risk** |  |

Table S2 Risk of bias assessment details of Lim et al 2024, using ROB2

| **Domain** | **Signalling question** | **Response** | **Comments** |
| --- | --- | --- | --- |
| **Bias arising from the randomization process** | 1.1 Was the allocation sequence random? | Y |  |
|  | 1.2 Was the allocation sequence concealed until participants were enrolled and assigned to interventions? | PY |  |
|  | 1.3 Did baseline differences between intervention groups suggest a problem with the randomization process? | N |  |
|  | **Risk of bias judgment** | **Low** |  |
| **Bias due to deviations from intended interventions** | 2.1. Were participants aware of their assigned intervention during the trial? | N |  |
|  | 2.2. Were carers and people delivering the interventions aware of participants' assigned intervention during the trial? | N |  |
|  | 2.3. If Y/PY/NI to 2.1 or 2.2: Were there deviations from the intended intervention that arose because of the experimental context? | N/A |  |
|  | 2.4 If Y/PY to 2.3: Were these deviations likely to have affected the outcome? | N/A |  |
|  | 2.5. If Y/PY/NI to 2.4: Were these deviations from intended intervention balanced between groups? | N/A |  |
|  | 2.6 Was an appropriate analysis used to estimate the effect of assignment to intervention? | Y |  |
|  | 2.7 If N/PN/NI to 2.6: Was there potential for a substantial impact (on the result) of the failure to analyse participants in the group to which they were randomized? | N/A |  |
|  | **Risk of bias judgment** | **Low** |  |
| **Bias due to missing outcome data** | 3.1 Were data for this outcome available for all, or nearly all, participants randomized? | Y |  |
|  | 3.2 If N/PN/NI to 3.1: Is there evidence that result was not biased by missing outcome data? | N/A |  |
|  | 3.3 If N/PN to 3.2: Could missingness in the outcome depend on its true value? | N/A |  |
|  | 3.4 If Y/PY/NI to 3.3: Is it likely that missingness in the outcome depended on its true value? | N/A |  |
|  | **Risk of bias judgment** | **Low** |  |
| **Bias in measurement of the outcome** | 4.1 Was the method of measuring the outcome inappropriate? | N |  |
|  | 4.2 Could measurement or ascertainment of the outcome have differed between intervention groups? | N |  |
|  | 4.3 Were outcome assessors aware of the intervention received by study participants? | PY |  |
|  | 4.4 If Y/PY/NI to 4.3: Could assessment of the outcome have been influenced by knowledge of intervention received? | PN |  |
|  | 4.5 If Y/PY/NI to 4.4: Is it likely that assessment of the outcome was influenced by knowledge of intervention received? | N/A |  |
|  | **Risk of bias judgment** | **Low** |  |
| **Bias in selection of the reported result** | 5.1 Were the data that produced this result analysed in accordance with a pre-specified analysis plan that was finalized before unblinded outcome data were available for analysis? | Y |  |
|  | 5.2 ... multiple eligible outcome measurements (e.g. scales, definitions, time points) within the outcome domain? | PN |  |
|  | 5.3 ... multiple eligible analyses of the data? | PN |  |
|  | **Risk of bias judgment** | **Low risk** |  |
| **Overall bias** | **Risk of bias judgment** | **Low risk** |  |

Table S3 Risk of bias assessment details of Heo et al 2024, using ROB2

| **Domain** | **Signalling question** | **Response** | **Comments** |
| --- | --- | --- | --- |
| **Bias arising from the randomization process** | 1.1 Was the allocation sequence random? | Y |  |
|  | 1.2 Was the allocation sequence concealed until participants were enrolled and assigned to interventions? | PY |  |
|  | 1.3 Did baseline differences between intervention groups suggest a problem with the randomization process? | N |  |
|  | **Risk of bias judgment** | **Low** |  |
| **Bias due to deviations from intended interventions** | 2.1. Were participants aware of their assigned intervention during the trial? | N |  |
|  | 2.2. Were carers and people delivering the interventions aware of participants' assigned intervention during the trial? | N |  |
|  | 2.3. If Y/PY/NI to 2.1 or 2.2: Were there deviations from the intended intervention that arose because of the experimental context? | N/A |  |
|  | 2.4 If Y/PY to 2.3: Were these deviations likely to have affected the outcome? | N/A |  |
|  | 2.5. If Y/PY/NI to 2.4: Were these deviations from intended intervention balanced between groups? | N/A |  |
|  | 2.6 Was an appropriate analysis used to estimate the effect of assignment to intervention? | Y |  |
|  | 2.7 If N/PN/NI to 2.6: Was there potential for a substantial impact (on the result) of the failure to analyse participants in the group to which they were randomized? | N/A |  |
|  | **Risk of bias judgment** | **Low** |  |
| **Bias due to missing outcome data** | 3.1 Were data for this outcome available for all, or nearly all, participants randomized? | Y |  |
|  | 3.2 If N/PN/NI to 3.1: Is there evidence that result was not biased by missing outcome data? | N/A |  |
|  | 3.3 If N/PN to 3.2: Could missingness in the outcome depend on its true value? | N/A |  |
|  | 3.4 If Y/PY/NI to 3.3: Is it likely that missingness in the outcome depended on its true value? | N/A |  |
|  | **Risk of bias judgment** | **Low** |  |
| **Bias in measurement of the outcome** | 4.1 Was the method of measuring the outcome inappropriate? | N |  |
|  | 4.2 Could measurement or ascertainment of the outcome have differed between intervention groups? | N |  |
|  | 4.3 Were outcome assessors aware of the intervention received by study participants? | PN |  |
|  | 4.4 If Y/PY/NI to 4.3: Could assessment of the outcome have been influenced by knowledge of intervention received? | N/A |  |
|  | 4.5 If Y/PY/NI to 4.4: Is it likely that assessment of the outcome was influenced by knowledge of intervention received? | N/A |  |
|  | **Risk of bias judgment** | **Low** |  |
| **Bias in selection of the reported result** | 5.1 Were the data that produced this result analysed in accordance with a pre-specified analysis plan that was finalized before unblinded outcome data were available for analysis? | PY |  |
|  | 5.2 ... multiple eligible outcome measurements (e.g. scales, definitions, time points) within the outcome domain? | PN |  |
|  | 5.3 ... multiple eligible analyses of the data? | PN |  |
|  | **Risk of bias judgment** | **Low risk** |  |
| **Overall bias** | **Risk of bias judgment** | **Low risk** |  |

Table S4 Risk of bias assessment details of Cho et al 2024, using ROB2
